# Evaluation of an Aqueous Extract from Horseradish Root (*Armoracia rusticana* Radix) against Lipopolysaccharide-Induced Cellular Inflammation Reaction

**DOI:** 10.1155/2017/1950692

**Published:** 2017-01-15

**Authors:** Corinna Herz, Hoai Thi Thu Tran, Melinda-Rita Márton, Ronald Maul, Susanne Baldermann, Monika Schreiner, Evelyn Lamy

**Affiliations:** ^1^Molecular Preventive Medicine, Institute of Prevention and Cancer Epidemiology, University Medical Center Freiburg, Elsässer Strasse 2, 79110 Freiburg, Germany; ^2^Department of Plant Quality, Leibniz Institute of Vegetable and Ornamental Crops Grossbeeren/Erfurt e.V., Echtermeyer-Weg 1, 14979 Grossbeeren, Germany

## Abstract

Horseradish (*Armoracia rusticana*) is a perennial crop and its root is used in condiments. Traditionally, horseradish root is used to treat bacterial infections of the respiratory tract and urinary bladder. The antiphlogistic activity, determined in activated primary human peripheral blood mononuclear cells (PBMC), was evaluated for an aqueous extract and its subfractions, separated by HPLC. Compound analysis was done by UHPLC-QToF/MS and GC-MS. The aqueous extract concentration-dependently inhibited the anti-inflammatory response to lipopolysaccharide (LPS) in terms of TNF-*α* release at ≥37 *μ*g/mL. Further, the cyclooxygenase as well as lipoxygenase pathway was blocked by the extract as demonstrated by inhibition of COX-2 protein expression and PGE_2_ synthesis at ≥4 *μ*g/mL and leukotriene LTB4 release. Mechanistic studies revealed that inhibition of ERK1/2 and c-Jun activation preceded COX-2 suppression upon plant extract treatment in the presence of LPS. Chemical analysis identified target compounds with a medium polarity as relevant for the observed bioactivity. Importantly, allyl isothiocyanate, which is quite well known for its anti-inflammatory capacity and as the principal pungent constituent in horseradish roots, was not relevant for the observations. The results suggest that horseradish root exerts an antiphlogistic activity in human immune cells by regulation of the COX and LOX pathway* via* MAPK signalling.

## 1. Introduction

Horseradish (*Armoracia rusticana*) belongs to the plant order Brassicales, family Brassicaceae. It is a perennial crop which is cultivated mainly in Europe and Asia. Particularly, its roots that are a rich source of biological active compounds are used in the diet as a condiment [1] due to their hot and piquant flavour and the penetrating smell. Horseradish root is also known for its anti-inflammatory and antibacterial characteristics and is consequently used for the treatment of acute sinusitis, bronchitis, and urinary bladder infection [2–5]. The characteristic hot flavour is mainly the result of the enzyme-mediated breakdown product called allyl isothiocyanate (allyl ITC) which is formed from the prodrug sinigrin [6]. Based on the current knowledge, it could be assumed that the health-promoting or curing effects of horseradish root are due to the bioactivity of allyl ITC. For this, strong anti-inflammatory activity was reported before [7]. Besides, the roots contain the antioxidant ascorbic acid [8] as well as the flavonoids kaempferol and quercetin [9] which are also known for their anti-inflammatory capacity.

So far, no scientific data are available which demonstrate the anti-inflammatory potency of horseradish root in a human cell based system or tried to clarify the relevance of allyl ITC for its bioactivity. We recently reported on the anti-inflammatory potential of nasturtium (*Tropaeolum majus nanum*) which also belongs to the plant order Brassicales [10]. In this study, we could not attribute the bioactivity of nasturtium to ITC formed from the plant. In the present study, the anti-inflammatory potential of aqueous horseradish root extracts as well as subfractions thereof, separated by HPLC, on lipopolysaccharide (LPS) and/or N-formyl-methionyl-leucyl-phenylalanine (fMLP) activated primary human peripheral mononuclear cells (PBMC) was studied. Inhibition of COX2/PGE_2_ as well as 5-LOX/LTB4 signalling pathway was the focus of the present study. In recent years, dual inhibitors of COX and LOX pathway have been considered as a new promising approach for the inhibition of inflammation with no or less side effects [11, 12].

## 2. Materials and Methods

### 2.1. Materials

Lymphoprep™ gradient was purchased from Progen (Heidelberg, Germany). RPMI 1640 medium, fetal calf serum, trypsin 10x (25 mg/mL), trypsin-EDTA 10x (5 mg/mL trypsin and 2.2 mg/mL EDTA), and phosphate buffered saline (PBS, without Ca and Mg) were from PAA Laboratories Gmbh (Coelbe, Germany). L-Glutamine, penicillin, and streptomycin were from Invitrogen (Karlsruhe, Germany). Camptothecin was from Tocris (Eching, Germany); Triton-X 100, milk powder, and N,N,N′,N′-tetramethyl-1-,2-diaminomethane (Temed) were from Carl Roth (Karlsruhe, Germany); DMSO, lipopolysaccharide (LPS), phorbol, 12-myristate 13-acetate, CFSE, ammonium persulfate, bovine serum albumin, ethanol absolute, hydrochloric acid (37%), leupeptin hemisulfate, p-coumaric acid, pepstatin A, Ponceau S, trypan blue, Tween 20, and ionomycin were from Sigma-Aldrich (Taufkirchen, Germany).

Antibodies against p-38 (Thr180/Tyr182), p-ERK1/2 (Thr202/Tyr204), p-JNK, and p-c-Jun (Ser73) and the horseradish peroxidase- (HRP-) labelled secondary antibodies, anti-mouse and anti-rabbit, were from Cell Signaling Technology (Boston, USA); anti-human COX-2 was from R&D Systems (Wiesbaden, Germany); anti-human COX-1 and anti-human 5-LOX (mouse monoclonal, clone 33) were from Santa Cruz Biotechnology (Heidelberg, Germany); and mAb against *β*-actin was from Sigma-Aldrich (Taufkirchen, Germany).

### 2.2. Isolation of Human PBMC and Cell Culture

The study was approved by the Ethical Committee of the University of Freiburg, Germany (EK-Freiburg: 597/14). Human PBMC were obtained with written consent from volunteers according to the guidelines of the local ethics committee. PBMC were isolated by centrifugation on a Lymphoprep gradient (density: 1.077 g/cm^3^, 20 min, 500 ×g). Cells were washed twice with prewarmed PBS and cell concentration and viability were determined using trypan blue. Fresh RPMI 1640 medium containing 10% heat-inactivated fetal calf serum, 2 mM L-glutamine, and 100 U/mL penicillin/streptomycin was added to PBMC (2 × 10^6^ cells/mL). Cells were treated either with solvent (10% water or 0.1% DMSO) or with aqueous plant extracts, washed twice with prewarmed PBS, and subsequently stimulated with 1 *μ*g/mL LPS and/or 1 *μ*M fMLP for different time points at 37°C in a humidified incubator with 5% CO_2_/95% air atmosphere. Subsequently, cells were washed with PBS and used for the bioassays as described below.

### 2.3. Plant Powder Extraction

Standardized lyophilized plant powder of the root of horseradish (*Armoracia rusticana *Radix, batch number PN19869) that is used in pharmacological remedy was provided by Repha GmbH (Langenhagen, Germany). Plant powder extraction was done as described before [10]. Briefly, one gram of plant powder was mixed with 10 mL double-distilled water or DMSO and directly incubated at 37°C for 30 min at 100 rpm. For the water extract, only glassware like vials, fennels, and flasks was used. The extract was strained through gauze and sterile filtered using a Millex syringe driven filter unit, 0.2 *μ*m (Merck Millipore, Darmstadt, Germany), and 6 serial dilutions were prepared in a 1 : 3 ratio. The initial concentration of the extracts was 33.33 mg/mL. 1 mL PBMC (1 × 10^6^ cells) suspension, prepared in a 24-well plate, was treated with 10 *μ*L of water extract. For the samples exposed to DMSO extract, 1 *μ*L was used in 1 mL cell suspension. The final concentration of DMSO in the cell suspension did not exceed 0.1%.

### 2.4. Separation of Plant Extracts Using Preparative HPLC

The separation of aqueous plant extracts using preparative HPLC was described before [10].

### 2.5. Nontargeted Analysis of Nonvolatile Metabolites by UHPLC-QToF-MS

The metabolites from 25 mg of finely powdered tissue were extracted with 250 *μ*L water at 37°C for 30 min. After this period, a defined volume of 70% methanol (70°C) (Carl Roth GmbH and Co. KG, Karlsruhe, Germany) was added. An aliquot of 100 *μ*L was used for the nontarget analysis of the metabolites [13]. In addition, fractions H1–H4 were analyzed concerning their metabolite profile. The aqueous plant extracts were filtered through a 0.2 *μ*m PTFE membrane (Amchro GmbH, Hattersheim, Germany) and analyzed with a 1290 Infinity UHPLC coupled with an Agilent 6250 QToF LC-MS (Agilent Technologies GmbH, Germany). Samples (5 *μ*L) were injected into a C18 column (2.1 × 50 mm, 1.8 *μ*m; Agilent Zorbax Entend-C18-Rapid Resolution HT). Column and sample temperatures were kept at 30°C and 10°C, respectively. The chromatographic gradient (solvent A: 0 min 98%, hold 3 min, 15 min 15%, 18 min 0%) was composed of 2 solutions (solvent A, 0.01% aqueous formic acid; solvent B 0.01% formic acid in acetonitrile) that were used to elute the compounds at a flow rate of 0.4 mL/min. An electron spray (ESI) source was used and spectra were collected in positive and negative ionization mode (acquisition rate: 1 spectrum/s) over a 100 to 1700* m/z* range (capillary voltage: 3.5 kV; source temperature: 300°C; nebulizer gas flow: 8 L/min at 35 psi, skimmer 65 V; fragmentor voltage: 175 V). For MS/MS experiments, spectra were collected over a 100 to 1700* m/z* range for MS and 50 to 900* m/z* for MS/MS selecting a maximum of 3 precursor ions per cycle. The collision energy was ramped with a slope of 4 and offset of 6. The raw data from the UHPLC-QToF-MS analysis were converted and processed by Mass Profiler Professional (MPP; Version 13.1, Agilent Technologies) using molecular feature extraction (MassHunter B.07.00) with the following settings: small molecules, minimum 500 counts for feature extraction and 5 000 counts for MPP analysis, ion species [M + H]^+^, [M − H]^−^, [M + Na]^+^, [M + NH_4_]^+^, [M + HCOO]^−^, and H_2_O as neutral loss, 15 ppm extraction width, and a quality score based on mass accuracy, isotope abundance, and isotope spacing of 80%. Raw data files were imported to MPP for recursive workflow. The formulas were then generated using the abovementioned ions and neutral loss with match tolerance of 20 ppm and 0.2 min.

The compounds were identified tentatively by comparing the mass spectra with MassHunter METLIN PCDL (Agilent, 79609 compounds) and public available databases for nonvolatile compounds.

### 2.6. Analysis of the Glucosinolate Profile of the Extract

The compounds were analyzed as previously reported [14].

#### 2.6.1. Nontargeted Analysis of Volatile Compounds Metabolites by Gas Chromatography-Mass Spectrometry

For the analysis of the volatile compounds, 500 *μ*L of water (HPLC grade, 37°C, Roth, Karlsruhe, Germany) was added to 50 mg plant material. A twister (stir bar sorptive extraction (SBSE)) device coated with polydimethylsiloxane (PDMS) (Gerstel GmbH & Co. KG, Mülheim an der Ruhr, Germany) was used to simultaneously trap the volatiles during the extraction. The suspension was kept at 37°C at 750 rpm for 30 min. The volatiles were analyzed with an Agilent 7010 Series gas chromatograph controlled by MassHunter (7.02, Agilent Technologies). The gas chromatograph was equipped with a BP5MS column (30 m × 250 *μ*m i.d., 0.25 *μ*m; SGE Analytical Sciences, VWR International GmbH, Darmstadt, Germany). The GC-MS with a Gerstel MPS 2 (Multiple Purpose Sampler, Gerstel GmbH & Co. KG, Mülheim an der Ruhr, Germany) injection system was operated with the following oven temperature program: 40°C for 3 min, increase of 2°C/min until 60°C and holding there for 2 min, and then increase of 3°C/min until 180°C. The carrier gas used was helium maintained at a constant flow rate of 1.2 mL/min. The cryofocusing program started at −100°C and then the temperature increased by 12°C/min to 280°C and was then maintained at 280°C for 3 min. Twisters™ desorption was performed with a Gerstel Thermal Desorption Unit (TDU, Gerstel GmbH & Co. KG, Mülheim an der Ruhr, Germany) with the following temperature program: starting temperature 25°C; increase of 100°C/min until 250°C, and holding for 4 min at 250°C. The MS analysis was carried out in a full-scan mode, with a scan range of* m/z* 50–300 and electron impact ionization energy of 70 eV.

#### 2.6.2. Determination of Cell Viability by Trypan Blue Exclusion Test

Cell viability was determined using the trypan blue exclusion test as described [15]. Isolated PBMC (2 × 10^6^ cells/mL) were pretreated with aqueous plant extracts (1–333 *μ*g/mL) or solvent control for 2 h and subsequently stimulated with 1 *μ*g/mL LPS for 36 h at 37°C in a humidified incubator with 5% CO_2_/95% air atmosphere. An aliquot of the cells of each sample was added to a 0.4% trypan blue solution (w/v), and viable cells were quantified in a Neubauer chamber.

### 2.7. Protein Analysis by Immunoblotting

Analysis of proteins by immunoblotting was performed as described before [16]. In brief, 15 *μ*g of protein was mixed with SDS-containing sample buffer and loaded onto acrylamide gel. Proteins were then transferred to a nitrocellulose membrane (Hybond ECL, GE Healthcare Life Sciences, Freiburg, Germany) using wet blotting (0.7 mA/cm^2^,  90 min). Membrane was blocked with 5% low fat milk in TBS/Tween 0.1% and incubated with primary antibody diluted with 5% low fat milk in TBS/Tween 0.1% or 5% BSA in TBS/Tween 0.1% for 1 h at RT or overnight at 4°C. Afterwards, horseradish peroxidase labelled secondary antibodies were incubated for 1 h at RT. Then, the signals were detected using ECL Advance Western Blotting Detection Kit (Hybond ECL, GE Healthcare Life Sciences, Freiburg, Germany) and the gel documentation system Molecular Imager® ChemiDoc™ XRS system (Bio-Rad, Munich, Germany). As loading control, the structural protein *β*-actin was detected on each membrane.

### 2.8. Quantification of TNF-*α* Release by ELISA Assay

To analyze TNF-*α* release, isolated PBMC (2 × 10^6^ cells/mL) were pretreated with aqueous plant extracts in the concentration of 1–333 *μ*g/mL for 3 h or solvent control (10% water) for 3 h and subsequently stimulated with 1 *μ*g/mL LPS for 3 h at 37°C in a humidified incubator with 5% CO_2_/95% air atmosphere. Supernatants were then used for photometric quantification of TNF-*α* using the TNF-*α* ELISA kit supplied from eBioscience (Frankfurt, Germany) according to the manufacturer's instructions.

### 2.9. Quantification of PGE_2_ Release by ELISA Assay

To quantify PGE_2_ release, isolated PBMC (2 × 10^6^ cells/mL) were analyzed with or without LPS stimulation for 24 h after pretreatment with the plant extracts (1–333 *μ*g/mL) or solvent control (10% water) for 6 h at 37°C in a humidified incubator with 5% CO_2_/95% air atmosphere. Supernatants were then used for photometric quantification of PGE_2_ using the PGE_2_ ELISA kit from R&D Systems (Wiesbaden, Germany) and Cayman (Hamburg, Germany) and were used for PGE_2_ quantification according to the manufacturer's instructions.

### 2.10. Quantification of LTB4 by ELISA Assay

To quantify LTB4 release, isolated PBMC (2 × 10^6^ cells/mL) were pretreated for 3 h with the plant extracts (333 *μ*g/mL) or subfraction H4, followed by 15 min incubation with 1 *μ*g/mL LPS and subsequently with 1 *μ*M fMLP for 15 or 30 min at 37°C in a humidified incubator with 5% CO_2_/95% air atmosphere. LTB4 release was photometrically quantified in the supernatants using the LTB ELISA kit from Cayman (Hamburg, Germany) according to the manufacturer's instructions.

### 2.11. COX-2 Enzyme Activity Assay

To analyze the direct inhibition of the COX-2 enzyme activity mediated by horseradish, the COX Inhibitor Screening Assay kit from Cayman (Hamburg, Germany) according to the manufacturer's instructions was used. Briefly, COX-2 enzyme was incubated with aqueous extract or fraction H4 for 10 min at 37°C and subsequently the reaction was initiated by adding arachidonic acid and incubating the mixture for 2 min at 37°C. Enzyme catalysis was stopped by adding saturated stannous chloride solution. Afterwards, PGF_2-*α*_ release was quantified using ELISA. As positive control, 25 or 50 *μ*M indomethacin was used.

### 2.12. Data Analysis

Data were analyzed using GraphPad Prism 5.0 software (La Jolla, CA). Data are presented as mean ± standard error of the mean (SEM) of at least three independent experiments and statistical significance was determined by the ordinary one-way ANOVA. *p* values < 0.05 (*∗*) were considered statistically significant and values <0.01 (*∗∗*) were considered highly statistically significant, compared to the respective controls.

## 3. Results

### 3.1. The Aqueous Extract from Horseradish Root Selectively Inhibits COX-2 Protein Expression and PGE_2_ Release but without Impacting COX-2 Enzyme Activity

**Figure 1:**
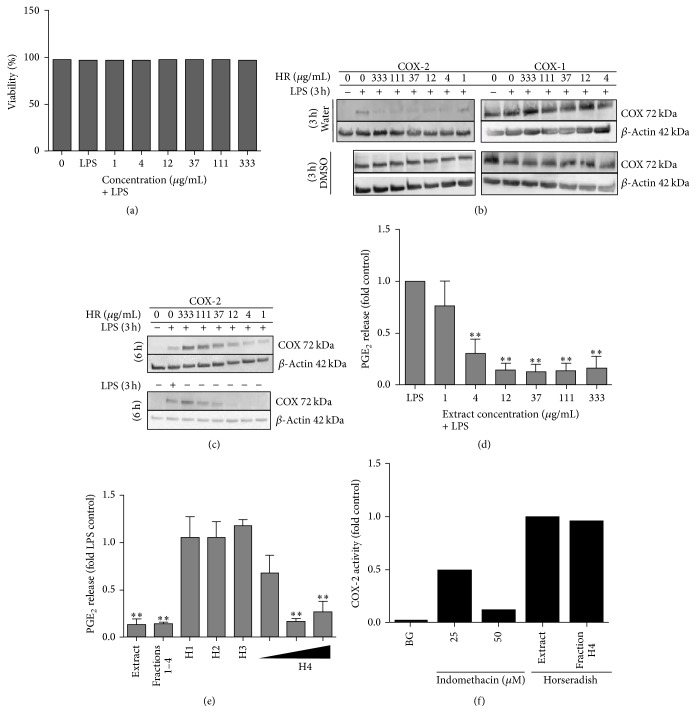
Efficacy of horseradish root on COX pathway activation in LPS stimulated human PBMC. (a) PBMC were stimulated with 1 *μ*g/mL LPS for 36 h after treatment with the aqueous plant extract for 2 h. Cell viability was determined by the trypan blue exclusion test. Bars are mean (*n* = 2). (b, c) PBMC were analyzed with or without LPS stimulation for 3 h after pretreatment with the plant extracts for 3 h. Total cell lysate was analyzed using immunoblotting. The figure shows representative immunoblots of COX-1 or COX-2. Membranes were probed with antibodies against *β*-actin which acted as loading control. (d) PBMC were analyzed with or without LPS stimulation for 24 h after pretreatment with the plant extracts for 6 h. Supernatants were then used for photometric quantification of PGE_2_ release using ELISA; bars are mean ± SEM (*n* = 5). (e) PBMC were pretreated with the complete aqueous extracts of horseradish or subfractions for 6 h followed by 18 h LPS stimulation (1 *μ*g/mL). Supernatants were used for quantification of PGE_2_ release. PGE_2_ release was calculated relative to the LPS stimulated control; bars are mean ± SEM (*n* = 3). (f) COX-2 was incubated with either indomethacin, the aqueous extract, or the subfraction H4. COX-2 enzyme activity was analyzed by quantification of PGF_2-*α*_ release using ELISA. PGF_2-*α*_ release was calculated relative to the LPS stimulated control; bars are mean (*n* = 2). ^*∗∗*^ *p* < 0.01. BG: background, that is, inactivated COX-2 enzyme plus inhibitor.

First, we analyzed whether the aqueous extract from horseradish root has toxic effects on human immune cells in the presence of LPS. For this, the trypan blue exclusion assay was used. None of the extract concentrations impaired the cell viability (Figure 1(a)) which ranged between 96.4 and 97.6% at all concentrations tested.

In an attempt to elucidate the anti-inflammatory potential of the plant, we studied its effect on the cyclooxygenase isoforms COX-1 and COX-2. Therefore, primary human PBMC were incubated with the aqueous extract at different concentrations prior to stimulation with LPS and subjected to immunoblot analysis of COX-1 and COX-2. Pretreatment of cells for 3 h strongly inhibited LPS-induced COX-2 expression at a broad concentration range from 1 *μ*g/mL to 333 *μ*g/mL (Figure 1(b)). To ascertain that the fraction of primarily water soluble compounds did account for the observed COX-2 regulation, a DMSO extract was additionally tested using the same experimental settings as described above but this had no impact on LPS stimulated COX expression (Figure 1(b)). This result indicated that the bioactive compounds were only present in the polar aqueous plant extract. The isoform COX-1, which is thought to be responsible for normal physiological functions in the human body, was not altered by any of the extracts tested (Figure 1(b)). In cells pretreated for 6 h with the aqueous extract and subsequent LPS treatment for 3 h, we found that higher concentrations (≥37 *μ*g/mL) stimulated COX-2 protein expression beyond the LPS effect (Figure 1(c)). To examine the contribution of the aqueous plant extract to this observation, we repeated the experiment in the absence of LPS. As shown in Figure 1(c), the extract induced COX-2 expression independent of LPS.

The enzyme COX-2 converts endogenous arachidonic acid to PGE_2_ in PBMC. The impact of horseradish on PGE_2_ release from LPS stimulated PBMC was next measured by ELISA. Despite the observed COX-2 induction by the aqueous plant extract after 6 h preincubation, our analysis demonstrated significant PGE_2_ inhibition in LPS-induced cells by the extract at >1 *μ*g/mL with a maximum of 87% at 37 *μ*g/mL (Figure 1(d)). To limit the number of potential relevant bioactive compounds, we then examined different subfractions prepared by HPLC fractionation. Only compounds from subfraction H4 accounted for the strong inhibitory effect on PGE_2_ release as observed by the whole plant extract (Figure 1(e)). The potency of H4 was then equal to the combination of all four fractions (Figure 1(e)). Next, we analyzed whether the aqueous extract directly inhibits COX-2 enzyme activity. However, neither the complete extract nor its bioactive subfraction H4 could block the activity of COX-2 enzyme (Figure 1(f)). In contrast, indomethacin, which was used as reference, inhibited COX-2 enzyme activity (Figure 1(f)).

### 3.2. The Aqueous Extract from Horseradish Inhibits LTB4 Release

**Figure 2:**
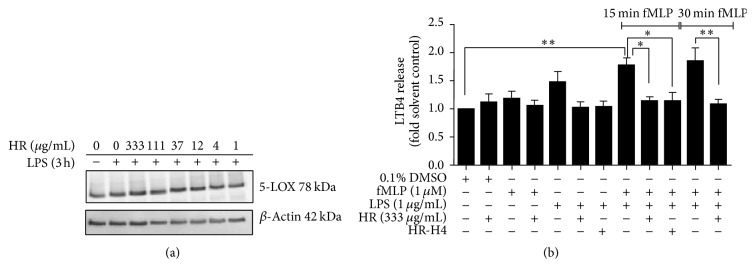
Effect of horseradish root on 5-LOX pathway activation. (a) PBMC were stimulated with 1 *μ*g/mL LPS for 6 h after pretreatment with the aqueous extract for 3 h. Total cell lysate was analyzed using immunoblotting. The picture shows representative blots of 5-LOX and *β*-actin (loading control). (b) 3 h treatment of PBMC with the plant extract was followed by 15 min incubation with 1 *μ*g/mL LPS and subsequently with 1 *μ*M fMLP for the indicated times. LTB4 release was photometrically quantified in the supernatants and calculated relative to the control; bars are mean ± SEM (*n* = 3). ^*∗*^ *p* < 0.05; ^*∗∗*^ *p* < 0.01.

To determine the effect on the 5-LOX pathway, 5-LOX protein expression was next studied. As shown in Figure 2(a), 5-LOX protein expression was not regulated at any extract concentrations tested. We then analyzed the impact of the aqueous extract and subfraction H4 on LTB4 release from PBMC, stimulated with LPS and fMLP. The cotreatment of cells with LPS and fMLP induced LTB4 release of 78% (15 min fMLP) and 86% (30 min fMLP) as compared to the solvent control. Pretreatment of cells with the whole aqueous extract or subfraction H4 significantly attenuated the release of LTB4 as stimulated by LPS/fMLP (63% for whole extract and 65% for H4 at 15 min fMLP and 77% for whole extract at 30 min fMLP) (Figure 2(b)).

### 3.3. The Aqueous Extract from Horseradish Interferes with LPS-Activated ERK Signalling

**Figure 3:**
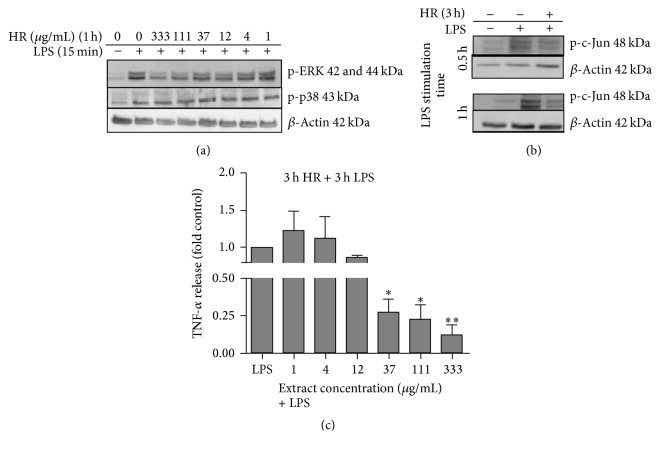
Effect of horseradish root on MAPK pathway activation and TNF-*α* release. PBMC were stimulated with 1 *μ*g/mL LPS for the indicated time points after treatment with the aqueous plant extract. (a, b) Total cell lysate was analyzed using immunoblotting. The picture shows representative blots of phosphorylated p-38 (Thr180/Tyr182) and ERK1/2 (Thr202/Tyr204) (a) or c-Jun (Ser73) (b). Blots were reprobed with antibodies against *β*-actin as loading control. (c) TNF-*α* was photometrically quantified in supernatants. The results were calculated relative to LPS stimulated cells; bars are mean ± SEM (*n* = 3). ^*∗*^ *p* < 0.05; ^*∗∗*^ *p* < 0.01.

The MAPK signalling pathway is one of the important early upstream cascades involved in inflammatory responses. Cells treated with the aqueous plant extract were therefore next subjected to analysis of MAPK activation. Concentration-dependent inhibition of LPS-triggered ERK1/2 activation was evident after exposure to the aqueous extract; the highest concentration (333 *μ*g/mL) almost completely inhibited ERK1/2 phosphorylation (Figure 3(a)). In contrast, the phosphorylation status of p38 was not altered by water extract treatment at any concentration tested. The activated form of ERK1/2 triggers transcriptional factors that alter the expression level of the target gene COX-2. In this, the transcription factor AP-1 plays a critical role in response to the inflammatory stimuli LPS. We therefore next investigated whether this transcription factor is blocked by pretreatment of LPS-activated PBMC with the aqueous extract. As depicted in Figure 3(b), pretreatment with the extract at 333 *μ*g/mL reduced the activation of c-Jun, the major component of AP-1, in a time-dependent manner.

Moreover, to assess whether the anti-inflammatory potential of horseradish also contributed to the inhibition of inflammatory cytokines, quantification of TNF-*α* was finally conducted. In Figure 3(c), it is shown that, similar to PGE_2_, the release of TNF-*α* from PBMC was also inhibited by whole extract treatment in a concentration-dependent manner. A concentration of ≥37 *μ*g/mL led to significant inhibition. At 333 *μ*g/mL, TNF-*α* release was inhibited by 87%.

### 3.4. Characterization of the Aqueous Extract of Horseradish Root Using UHPLC-QToF and GC-MS

**Figure 4:**
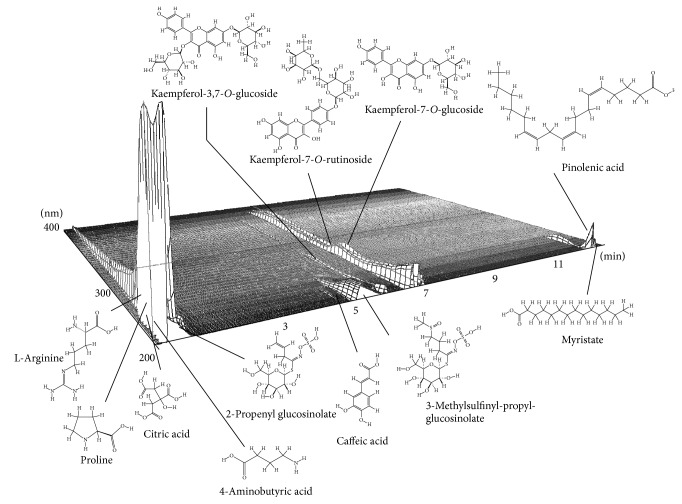
Metabolomic analysis of the aqueous plant extract using UHPLC-QToF-MS. The compounds were identified tentatively by comparing the mass spectra with MassHunter METLIN PCDL for the nonvolatile compounds and public available databases.

**Figure 5:**
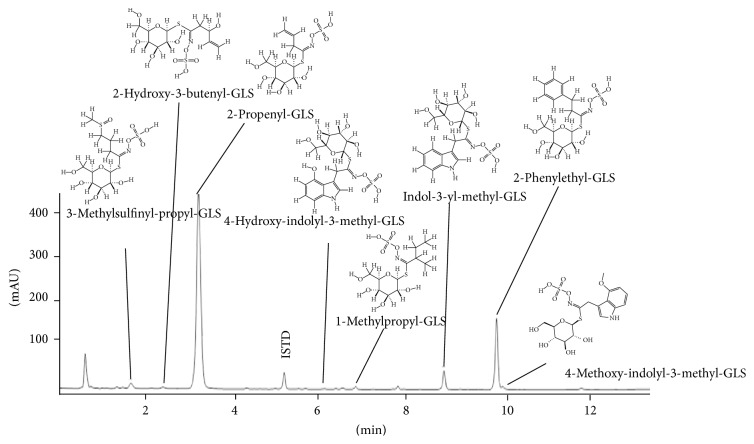
Glucosinolate profile of the aqueous extract of horseradish root. ISTD: internal standard; GLS: glucosinolate.

To characterize the whole aqueous plant extract and the subfractions, we performed metabolomic screening and a targeted analysis of glucosinolates (GLS) [14] of the aqueous plant extract. Prominent compounds in the extract were the amino acids arginine and proline, citric acid, phenolic compounds including caffeic acid and kaempferol derivatives, the main GLS, 2-propenyl-GLS and 3-methylsulfinyl-propyl-GLS, and fatty acid derivatives (Figure 4). Targeted analysis revealed 2-propenyl-GLS and 2-phenylethyl-GLS as major GLS derivatives (Figure 5). Analysis of subfractions H1–H4 revealed in fraction H1, for instance, GLS; in H2, kaempferol derivatives; in H3, caffeic acid and its derivatives as well as sugars; in H4, fatty acid and derivatives (Table 1).

**Figure 6:**
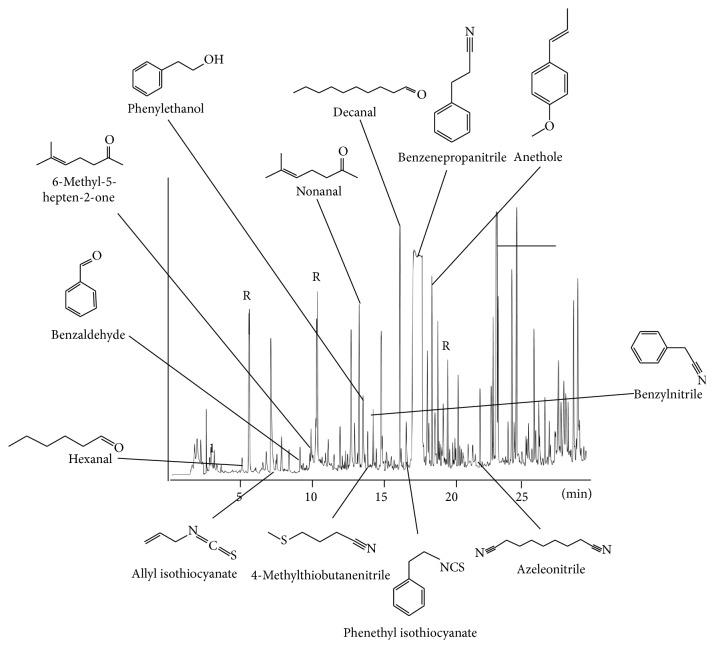
Nontargeted analysis of volatile compounds from the aqueous extract of horseradish root by GC-MS.

Additionally, we determined the volatile compounds present in the aqueous plant extract (Figure 6). We identified various glucosinolate derived volatiles including ITC, isocyanates, and nitriles; further compounds derived from the phenylpropanoid pathway (e.g., benzaldehyde and phenylethanol) or from fatty acids (e.g., hexanal and nonanal).

## 4. Discussion

In addition to its use as a spice, horseradish root is used in traditional medicine as a treatment against inflammatory diseases [2, 3]. However, so far, only few scientific data are available which investigated the bioactivity of this plant which could provide a rationale for its health benefit. The present study showed that an aqueous plant extract of horseradish root dually blocked the COX and 5-LOX pathway in primary human immune system.

The chemical analysis carried out in this study confirmed that horseradish is a rich source of GLS which are hydrolysed into allyl- and phenylethyl ITC. Earlier reports demonstrated for phenylethyl ITC that this could suppress COX-2 in LPS stimulated RAW macrophages [17] and recently it was shown that methanol extract of horseradish root has an anti-inflammatory activity in an LPS-stimulated murine macrophage cell line which was claimed to be possibly related to allyl ITC [18]. However, for the anti-inflammatory effects of horseradish as observed in the present study, ITC are not relevant. This was confirmed by us due to the absence of ITC in the bioactive fraction. A similar observation was made by us earlier using a water extract from nasturtium (*Tropaeolum majus nanum*), which also belongs to the plant order Brassicales [10]. According to the applied chromatographic separation system, it can be assumed that the target substances in the active extract possess a medium polarity. Fatty acids such as linoleic acid were contained in this fraction. Linoleic acid was earlier found to reduce the LPS-mediated inflammatory response in human monocytic THP-1 cells in terms of blocking the secretion of interleukin-6 (IL-6), IL-1beta, and TNF-*α* [19]. However, at present, it remains unclear which of the compounds is responsible for the bioactivity observed in the cell assay experiments. Further efforts are necessary to identify these.

In our study, a differential effect on COX-2 protein expression was observed, dependent on the pretreatment time with the aqueous plant extract. But, despite the stimulatory effect of COX-2 at longer incubation periods, this was not associated with an increase in PGE_2_ levels. Plant compounds like ajoene from garlic [20] or alkamides from* Echinacea* [21] all have been reported to increase LPS-induced COX-2 mRNA and/or protein expression while concomitantly inhibiting PGE_2_ formation. In these studies, this was due to direct inhibition of COX-2 enzyme activity, a mechanism common to nonsteroidal anti-inflammatory drugs such as ibuprofen or acetylsalicylic acid. Acetylsalicylic acid, for example, inhibits COX by acetylation of an essential serine at the active enzyme site [22]. In our study, however, neither the whole extract nor a subfraction could block the COX-2 enzyme directly. Prostanoids are suggested to be negative feedback regulators of COX-2 expression so inhibition of PGE_2_ synthesis may lead to increased COX-2 expression as suggested before [20]. Early phase and selective inhibition of the MAPK ERK1/2 was observed by cell treatment with horseradish extracts. These results suggest that inhibition of the arachidonic acid pathway by the plant happens at early upstream signals. Moreover, horseradish inhibited the phosphorylation of c-Jun which points at the importance of ERK1/2-AP1 pathway inhibition for the observed anti-inflammatory activity. Further, this interference could also account for the inhibitory effect of horseradish on TNF-*α* since it has been shown that blocking either the JNK, p38 MAPK, or ERK pathway is sufficient to block induction of TNF-*α* by LPS.

## 5. Conclusion

In conclusion, this study provides evidence in a human cell based system of the therapeutic efficacy of horseradish root preparations in inflammation-mediated events. The dual inhibition of the AA metabolism found here underlines its potential usefulness, as side effects might be less severe compared to selective COX blockers. Studies are now needed to clarify the relevance of the* in vitro* results at hand for the situation in patients. Also, further efforts should be made to identify the relevant bioactive components in horseradish and assess their bioavailability. The effects were not mediated by ITC. ITC are known potent anti-inflammatory agents and may account for a variety of bioactive effects observed in Brassicales plants. However, this study clearly demonstrates that it is not always the characteristic phytochemical class of Brassicales that explains bioactivity.

## Figures and Tables

**Table 1:** Tentatively identified compounds in subfractions H1 to H4 by UHPLC-QToF.

Compound name	Fraction	Formula	Mass	Error [ppm]
**2-Propenyl-GLS**	H1	C_10_ H_17_ N O_9_ S_2_	359.035	1.2
**3-Methylsulfinyl-propyl-GLS**	H1	C_11_ H_21_ N O_10_ S_3_	423.033	0.5
**2-Phenylethyl-GLS**	H1	C_15_ H_21_ N O_9_ S_2_	423.0568	0.5

**Kaempferol-7-*O*-rutinoside**	H2	C_27_ H_30_ O_15_	594.1585	0.2
Kaempferol-7-*O*-glucoside	H2	C_21_ H_20_ O_11_	448.1006	0.3

Caffeic acid	H3	C_9_ H_8_ O_4_	180.0423	2.2
**Coumaroyl quinic acid**	H3	C_16_H_18_O_8_	338.1002	6.5
Galloylglucose	H3	C_13_H_16_O_10_	332.0743	6.6

**Linoleic acid**	H4	C_18_ H_32_ O_2_	280.240	0.7
**Eicosanedioic acid**	H4	C_20_ H_38_ O_4_	342.278	2.9
**13-Hexadecenoic acid**	H4	C_16_ H_30_ O_2_	254.225	1
**3*S*-Hydroxypalmitic acid**	H4	C_16_ H_32_ O_3_	272.235	0.4
**9,10-Dihydroxyhexadecanoic acid**	H4	C_16_ H_32_ O_4_	288.230	1.8
**9*R*,10*S*-Epoxy-12*Z*-octadecenoic acid**	H4	C_18_ H_32_ O_3_	296.235	0.5
**17-Hydroxy-9Z-octadecenoic acid**	H4	C_18_ H_34_ O_3_	298.251	0.6
**14-Hydroxystearic acid**	H4	C_18_ H_36_ O_3_	300.266	1.4
**10,11-Dihydroxystearic acid**	H4	C_18_ H_36_ O_4_	316.261	2.1
**C16 Sphingosine**	H4	C_16_ H_33_ N O_2_	271.251	3.6
**PI(18:2/20:5)**	H4	C_47_ H_77_ O_13_ P	880.510	1.2
**PI(18:0/0:0)**	H4	C_27_ H_53_ O_12_ P	600.327	0.4
**PG(17:2/14:1)**	H4	C_37_ H_67_ O_10_ P	702.447	1.3
**PG(16:0/0:0)**	H4	C_22_ H_45_ O_9_ P	484.280	2

PI: phosphatidylinositol; PG: phosphatidylglycerol.
